# ZPBP and fertility-associated proteomes as novel biomarkers: A shotgun proteomics approach in Murrah buffalo (*Bubalus bubalis*) bulls’ seminal plasma and cryopreserved spermatozoa

**DOI:** 10.1371/journal.pone.0333272

**Published:** 2025-10-22

**Authors:** Hikmayani Iskandar, Syahruddin Said, Tulus Maulana, Ekayanti Mulyawati Kaiin, Berlin Pandapotan Pardede, Isyana Khaerunnisa, Widya Pintaka Bayu Putra, Raden Iis Arifiantini, Fuad Hasan, Göran Andersson, Sarmin Sarmin, Claude Mona Airin, Reski Amalia, Pudji Astuti

**Affiliations:** 1 Research Center for Applied Zoology, National Research and Innovation Agency (BRIN), Bogor, Indonesia; 2 Divisions of Veterinary Reproduction and Obstetrics, School of Veterinary Medicine and Biomedical Sciences, IPB University, Bogor, Indonesia; 3 Department of Animal Science, Faculty of Agriculture, Universitas Sumatera Utara, Medan, Indonesia; 4 Department of Animal Biosciences, Swedish University of Agricultural Sciences, Uppsala, Sweden; 5 Department of Physiology, Faculty of Veterinary Medicine, Universitas Gadjah Mada, Yogyakarta, Indonesia; The Islamic University, IRAQ

## Abstract

Comprehensive identification of seminal plasma and spermatozoa proteins is essential to understand sperm biology and the molecular mechanisms underlying bull fertility. In this study, we conducted a quantitative proteomic analysis of seminal plasma and cryopreserved spermatozoa from Murrah buffalo (*Bubalus bubalis*) bulls using a comparative shotgun proteomics approach to identify proteins associated with reproduction. 1,893 and 1,913 proteins were identified in seminal plasma and spermatozoa, respectively. Among these, 232 proteins were selectively identified in both seminal plasma and spermatozoa. Notably, zona pellucida binding protein (ZPBP) was explicitly expressed in spermatozoa, while no reproduction-associated proteins were detected in the seminal plasma proteome. Gene ontology (GO) analysis revealed that most proteins were involved in reproduction-related cellular and metabolic processes. Protein–protein interaction (PPI) network analysis further indicated that ZPBP is involved in biological processes, including acrosome formation and spermatid development. ZPBP was identified with a low false discovery rate (FDR = 0.0021), underscoring its statistical significance. Several proteins were associated with key reproductive functions, including spermatogenesis, sperm motility, energy metabolism, and cellular stress responses. These findings reveal distinct proteomic signatures with strong potential as candidate biomarkers for assessing bull fertility, supporting more accurate reproductive evaluations, and the strategic selection of genetically and reproductively superior breeding bulls in buffalo reproductive management programs.

## 1. Introduction

Over the past decade, buffalo breeding has grown significantly, driven by increasing market demand and greater consumer acceptance of buffalo-derived products. Amidst the global demand for animal protein and mounting pressure on cattle populations, buffaloes have emerged as a strategic alternative for sustainable livestock production. In many countries, buffaloes serve as a primary source of milk; however, their full productive potential remains underutilized, primarily due to the low reproductive efficiency of breeding bulls, particularly evidenced by low conception rates following artificial insemination [1,2]. In particular, Murrah buffalo (*Bubalus bubalis*) are highly regarded for their adaptability to tropical climates, resilience to disease, and firm dairy productivity. This high-yielding breed holds considerable genetic potential for improving productivity and sustainability in large ruminant systems [3–7]. Despite advancements in reproductive technologies and diagnostic tools, accurately predicting bull fertility remains a significant challenge. Conventional semen evaluation parameters, such as motility and morphology assessments, have shown limited predictive power for accurately determining fertility potential in breeding bulls [8,9]. This limitation has driven the search for molecular and functional biomarkers that can provide more reliable indicators of reproductive potential.

Proteomic profiling of seminal plasma and spermatozoa from bulls with varying fertility statuses offers a promising approach for identifying differentially expressed proteins associated with reproductive traits. These proteins may serve as candidate molecular markers for understanding the mechanisms of spermatogenesis, assessing sperm quality, and enhancing the accuracy of fertility predictions [10]. Recent advances in proteomics have enabled the characterization of reproductive tissues and fluids with greater molecular detail, revealing protein signatures strongly correlated with reproductive performance [11–13]. While proteomic approaches have been extensively applied in *Bos taurus* to study reproductive physiology, semen quality, and embryonic development [8,14–16], similar efforts in buffalo remain limited. Buffalo bull spermatozoa are notably more susceptible to cryopreservation-induced damage, compromising both their structural integrity and post-thaw function, leading to lower fertility rates than cattle [9]. Although some studies have explored buffalo reproductive traits, thermal stress responses [17], seminal plasma composition [18], and broader biological processes [19], investigations into fertility-associated sperm proteins in buffalo remain relatively underdeveloped. Limited proteomic studies using 2D-DIGE, MALDI-TOF, and Liquid chromatography-tandem mass spectrometry (LC-MS/MS) have identified only a small set of differentially expressed proteins related to fertility outcomes in buffalo bulls [3,20,21].

Proteomics has thus emerged as a powerful tool in andrological research, offering a molecular-level perspective on the complex processes governing spermatozoa function and male fertility [21]. The analysis of the spermatozoa proteome enables comprehensive identification of key proteins involved in sperm maturation, capacitation, acrosome reaction, and fertilization [22]. High-throughput proteomic technologies have facilitated the discovery of seminal plasma and spermatozoa proteins differentially expressed between high- and low-fertility bulls, many of which are linked to motility, membrane integrity, and fertilization competence [3]. These fertility-associated proteins hold great potential as reliable biomarkers and could be developed into molecular tools for more precise fertility evaluation in breeding bulls. This study aims to define the fertility-associated proteome of Murrah buffalo bulls through a comprehensive comparative proteomic analysis of seminal plasma and cryopreserved spermatozoa. By identifying specific proteins associated with fertility traits, this research contributes to developing reliable molecular biomarkers for assessing bull reproductive competence. Ultimately, the outcomes of this study are expected to improve the precision of fertility assessment and support the strategic selection of genetically and reproductively superior buffalo bulls, thereby enhancing the efficiency, sustainability, and success of buffalo breeding programs in the future.

## 2. Materials and methods

### 2.1. Ethical approval

The animal ethics committee of the National Research and Innovation Agency (BRIN), Indonesia, reviewed and approved all experimental procedures involving animals under approval number 049/KE.02/SK/03/2023. This approval pertains to animal models and the experimental design employed in the present study.

### 2.2. Sample collection, processing, and protein extraction

Fresh semen samples were collected from eight Murrah buffalo bulls (*Bubalus bubalis*), aged 7–10 years, maintained at the Regional Artificial Insemination Center (RAIC), Sumatra, Indonesia. Semen collection, spermatozoa separation, and cryopreservation were performed by trained AI Center staff under veterinary supervision, following established operational standards and animal welfare guidelines. For each bull, semen was collected three times using an artificial vagina in accordance with AI Center procedures. Immediately after collection, semen quality was assessed macroscopically and microscopically. Only ejaculates meeting the following criteria were included: progressive sperm motility ≥70% and sperm concentration ≥800 × 10⁶ cells/mL. For seminal plasma preparation, 2 mL of each qualified semen sample was centrifuged at 3,000 × g for 30 min to separate spermatozoa from seminal plasma. The supernatant was collected and stored at –20°C until proteomic analysis. Cryopreserved semen was prepared using the Center’s standard freezing protocol. All procedures up to semen freezing and seminal plasma storage were conducted exclusively by AI Center personnel. The research team received only the commercially prepared frozen semen and seminal plasma for downstream proteomic analysis.

For spermatozoa protein extraction, frozen semen samples were thawed at 37°C for 30 s and washed three times with phosphate-buffered saline (PBS) by centrifugation at 1,800 × g for 10 min to remove residual seminal plasma. The resulting sperm pellet contained approximately 1–2 × 10⁸ cells per sample. Proteins were extracted using PRO-PREP™ protein extraction solution (iNtRON Biotechnology, Seongnam, Korea), following the manufacturer’s instructions. Briefly, 400 µL of extraction buffer was added to the pellet, followed by incubation at –20°C for 20 min. Lysates were centrifuged at 10,000 × g for 5 min at 4°C, and the soluble protein supernatant was collected.

**Fig 1:**
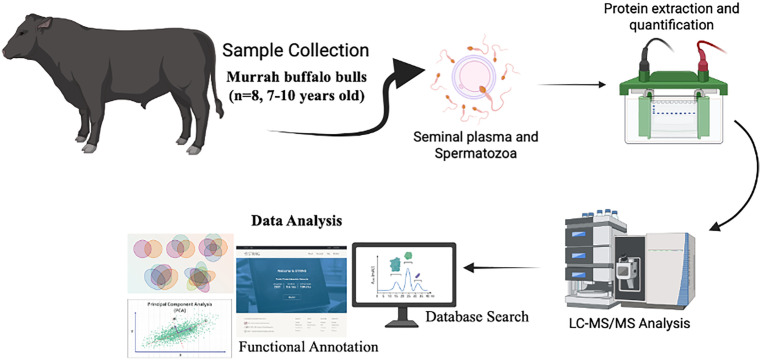
Schematic workflow for the proteomic analysis of Murrah buffalo bulls. The illustration was created with BioRender (www.biorender.com).

Protein concentrations from seminal plasma and spermatozoa fractions were quantified using the bicinchoninic acid (BCA) assay (Pierce™ BCA Protein Assay Kit 23225, Thermo Scientific, Rockford, IL, USA). Equal amounts of protein (50 µg) were used for all subsequent SDS-PAGE and proteomic analyses to ensure consistency and comparability across samples (Fig 1).

### 2.3. SDS-PAGE and protein visualization

Frozen semen samples were thawed in a water bath at 37°C for 30 sec, followed by three consecutive washes with PBS to remove cryoprotectants and extender residues. The samples were then centrifuged at 1,800xg to obtain spermatozoa pellets. According to the manufacturer’s instructions, total protein was extracted from the pellets using PRO-PREP™ protein extraction solution (iNtRON Biotechnology, Korea). Protein concentrations were determined using the BCA assay to ensure equal loading before electrophoresis. For protein separation, sodium dodecyl sulfate-polyacrylamide gel electrophoresis (SDS-PAGE) was conducted using a 12% polyacrylamide precast gel (ExpressPlus, M01210; Genscript Biotech Corp., Hong Kong) under denaturing conditions at a constant voltage of 140 V and 75 mA for 55 min. After electrophoresis, proteins were visualized by staining with coomassie brilliant blue (CBB). A pre-stained broad-range protein ladder (M00624, Genscript Biotech Corp) with a molecular weight range of 5–270 kDa was included in each run to facilitate molecular size determination. The relative intensities of protein bands were quantified using ImageJ software (National Institutes of Health, USA) for comparative analysis among samples.

### 2.4. In-gel tryptic digestion and peptide preparation

Distinct protein bands were excised from the SDS-PAGE gel and subjected to in-gel digestion. Gel slices were destained with 200 µL of a solution containing ammonium bicarbonate (80 mg) in equal parts of acetonitrile (ACN) and ultrapure H_2_O, incubated at 37°C for 30 min, and the process was repeated twice. Reduction was carried out with 30 µL of reducing buffer (a total of 3.3 µL TCEP (Tris(2-carboxyethyl)phosphine) was added to 300 µL digestion buffer containing 10 mg ammonium bicarbonate dissolved in 5 mL ultrapure H_2_O) at 60°C for 10 min. Alkylation was followed by adding 30 µL of iodoacetamide in the digestion buffer, and the mixture was incubated in the dark at room temperature for 1 hour. The samples were washed twice with destaining solution at 37°C for 15 min each. To prepare for digestion, gel pieces were treated with 50 µL of 100% ACN for 15 min and briefly air-dried. Trypsin digestion was initiated with 50 µL of digestion buffer containing activated trypsin (10 ng/µL) and incubated at 37°C for 4 hours.

### 2.5. Peptide fractionation and LC-MS/MS analysis

Dried peptides were reconstituted in 50 µL of dissolving solution (2% ACN, 98% ultrapure H_2_O, and 0.1% formic acid) and centrifuged at 10,000xg for 10 min. A 2.5 µL aliquot of each sample was injected into a Nano LC Ultimate 3000 system coupled with a Q exactive plus orbitrap high-resolution mass spectrometer (Thermo Scientific). Peptides were first trapped using a 30 µm x 5 mm trap column (Thermo Scientific 164649) and then separated on a PepMap RSLC C18 analytical capillary column (75 µm x 15 cm, 3 µm particle size, 100 Å pore size; Thermo Scientific ES800) at a flow rate of 300 nL/min. The elution gradient was as follows: 2–35% solvent B over 27 min, 35–99% B over 10 min, 99% B for 15 min, followed by re-equilibration at 2% B for 30 min. Solvent A consisted of water with 0.1% formic acid, and solvent B consisted of ACN with 0.1% formic acid. MS data were acquired over an m/z range of 200–2000 using the LTQ-orbitrap mass analyzer.

### 2.6. Database search, chemometric, and bioinformatic analysis

The LC-MS/MS raw spectral data (input) were processed using *Proteome Discoverer v2.2* (Thermo Scientific) using the Sequest high-throughput (HT) search algorithm. Following refinement based on unique protein counts, Sequest HT scores, and recovery rates, 311 proteins were selected from seminal plasma and 260 from sperm for further analysis. The output of this step was a list of peptide-spectrum matches (PSMs), which were subsequently filtered by applying a false discovery rate (FDR) threshold of 1% (strict) and 5% (relaxed). Only proteins with an HT score greater than zero and supported by at least two unique peptides, within a precursor mass tolerance of 10 ppm, were retained as valid identifications (output). Validated protein identifications were then mapped against the *Bos taurus* reference proteome database (UniProt, https://www.uniprot.org/proteomes/), from which accession numbers and protein identifiers were retrieved (output). These identifiers were used as input for downstream analysis.

A false discovery rate (FDR) of 1% (strict) and 5% (relaxed) was applied. Protein identifications required an HT score greater than zero and at least two unique peptides with a precursor mass tolerance of 10 ppm. For functional characterization, the list of identified proteins (input) was analyzed using the PANTHER classification system (http://pantherdb.org/) and the database for annotation, visualization, and integrated discovery (DAVID v6.8; https://david.ncifcrf.gov), producing outputs in the form of gene ontology (GO) terms, molecular functions, biological processes, and pathway classifications. Comparative analysis of protein distribution across groups was performed using a web-based Venn diagram generator (https://bioinformatics.psb.ugent.be/webtools/Venn/), with the input being sets of protein identifiers and the output visualizing shared and unique proteins between experimental groups. Protein-protein interaction (PPI) networks were constructed using the search tool for the retrieval of interacting genes/proteins (STRING) database (v11.0) (http://string-db.org). Uniprot accession numbers derived directly from the LC-MS/MS dataset (input) were uploaded into STRING, which generated PPI networks (output), including predicted interactions, confidence scores, and functional clusters.

## 3. Results

**Fig 2:**
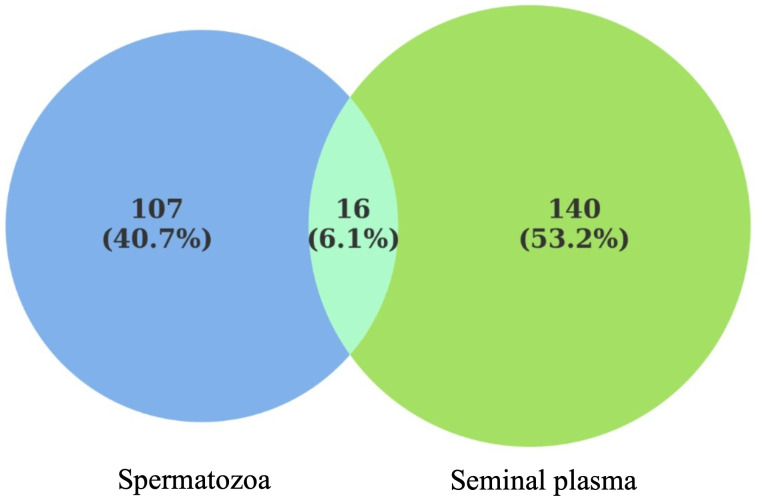
Venn diagram of seminal plasma and spermatozoa proteins in Murrah buffalo bulls. The proteomic distribution reveals a pronounced compartmentalization between seminal plasma and spermatozoa, with 140 and 107 proteins uniquely identified in each fraction, respectively, and only 16 proteins co-localized, underscoring a pronounced compartmentalization of protein functions.

Using a high-resolution LC-MS/MS platform, 1,893 proteins were identified in seminal plasma and 1,913 proteins in spermatozoa collected from eight Murrah buffalo bulls. Venn diagram analysis (Fig 2) revealed that the proteomes of seminal plasma and spermatozoa are largely distinct, with 140 proteins (53.2%) uniquely identified in seminal plasma, 107 proteins (40.7%) uniquely identified in spermatozoa, and only 16 proteins (6.1%) between both fractions.

**Fig 3:**
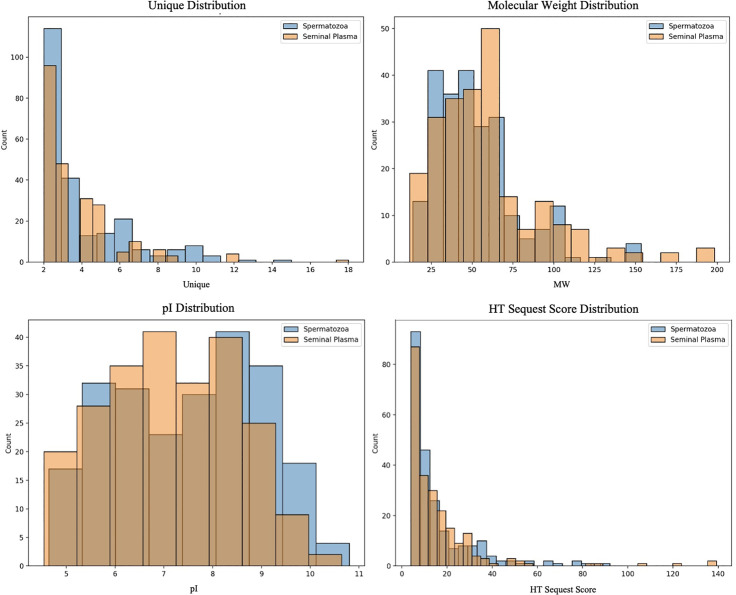
Distribution of molecular weight, pI values, and unique peptides of identified proteins in Murrah buffalo bulls spermatozoa and seminal plasma. The proteomic distribution profiles demonstrate that proteins from both fractions are predominantly characterized by low molecular weight, low peptide count, and acidic to neutral isoelectric points. Notably, seminal plasma proteins exhibit a tendency toward higher molecular weights and more acidic pI values relative to spermatozoa proteins, underscoring the distinct molecular composition and specialized functional roles of each compartment within the bull reproductive system.

The histograms illustrate the proteomic characteristics of seminal plasma and spermatozoa (Fig 3). The distribution of unique peptides indicates that most proteins in both sample types are detected at low frequencies, with spermatozoa exhibiting a slightly broader range of unique peptide counts. Molecular weight distributions were broadly similar between the two groups, although seminal plasma contains a somewhat higher proportion of proteins in the 50–75 kDa range. Regarding isoelectric point (pI), proteins in both fractions span a broad spectrum, but seminal plasma shows a greater clustering around neutral pI values (7–8). The HT sequest score distributions suggest comparable protein identification confidence across samples, with spermatozoa displaying a few proteins with higher scores. Overall, the proteomic profiles of seminal plasma and spermatozoa were broadly similar, with subtle differences in protein abundance, molecular weight, and pI distribution.

**Fig 4:**
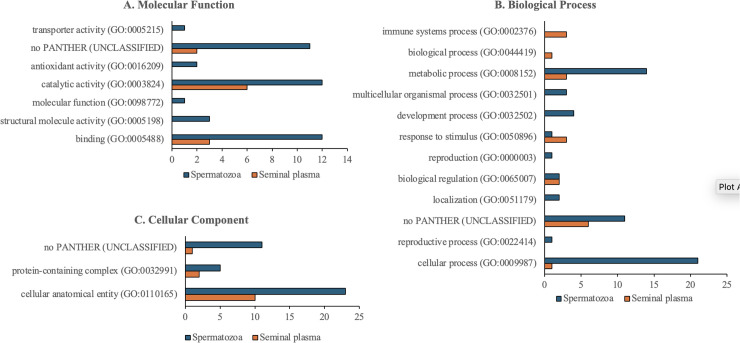
Comparison of proteome functions in seminal plasma and spermatozoa of Murrah buffalo bulls: (A) Molecular functions, (B) Biological processes, (C) Cellular components. The GO classification reveals that spermatozoa proteins in Murrah buffalo are predominantly involved in catalytic and binding functions, reproductive and metabolic processes, and localized within complex cellular structures, while seminal plasma proteins exhibit limited functional diversity and are primarily associated with support roles.

As shown in Fig 4, the functional annotation using the PANTHER GO classification system revealed that many proteins remained unclassified, likely due to the limited availability of *Bubalus bubalis* proteomic data, necessitating reliance on *Bos taurus* as the reference species. In terms of molecular function, the majority of proteins in both seminal plasma and spermatozoa were involved in binding activity (GO:0005488) and catalytic activity (GO:0003824) (Fig 4A). Biological process analysis identified cellular processes (GO:0009987) and metabolic processes (GO:0008152) as dominant pathways, with additional relevance to reproduction (GO:0000003) and reproductive processes (GO:0022414) (Fig 4B). The most common cellular component classification was cellular anatomical entity (GO:0110165), with spermatozoa proteins predominantly localized to complex intracellular structures, whereas seminal plasma proteins were mainly associated with extracellular regions (Fig 4C).

**Fig 5:**
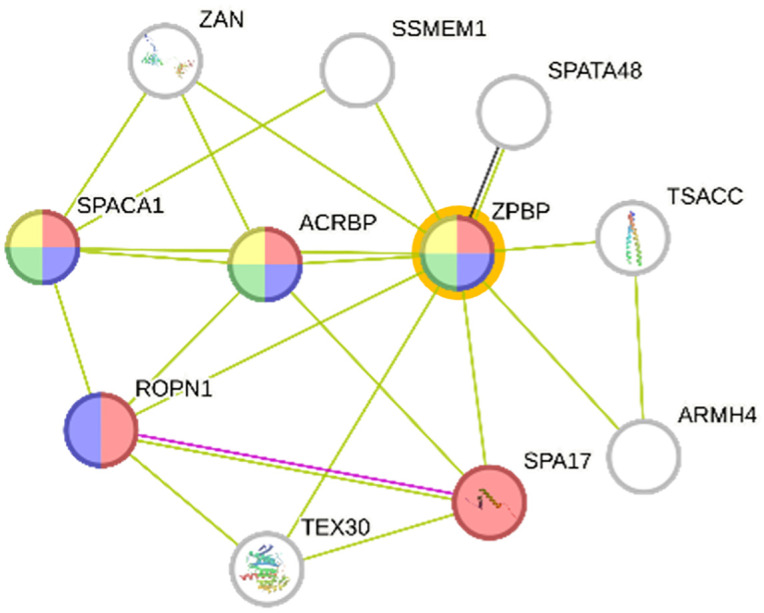
Interaction analysis of Murrah buffalo bulls’ spermatozoa proteins. ZPBP (STRING platform: http://string-db.org. The protein-protein interaction network highlights ZPBP as a central node with functional associations to acrosomal and sperm-binding proteins such as ACRBP, SPACA1, and SPA17, underscoring its pivotal role in sperm-oocyte recognition and fertilization processes.

**Fig 6:**
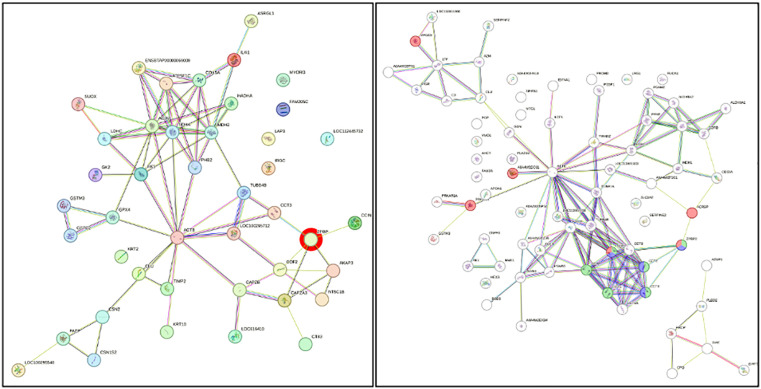
Interaction analysis of spermatozoa (A) and seminal plasma (B) proteins in Murrah buffalo bulls (STRING platform: http://string-db.org). The interaction networks reveal distinct functional modules, with spermatozoa (A) predominantly centered around cytoskeletal components such as ACTB, suggesting key roles in structural organization and intracellular transport. In contrast, panel seminal plasma (B) is enriched in molecular chaperones and translational machinery, indicative of essential processes involved in protein folding, stability, and sperm maturation support.

**Fig 7:**
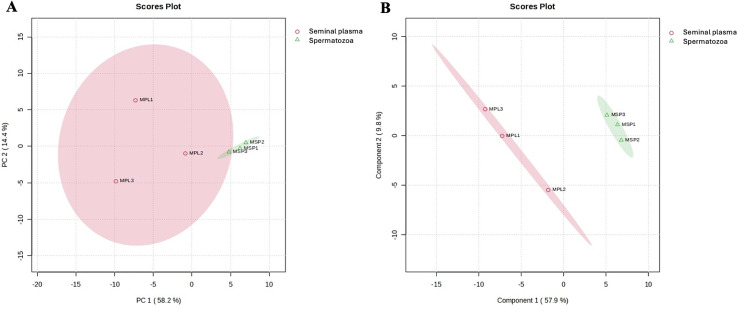
Protein profiles of seminal plasma and spermatozoa in Murrah buffalo bulls. **(A)** Principal component analysis and **(B)** Partial least squares discriminant analysis. Multivariate analysis using PCA and PLS-DA reveals a clear proteomic distinction between spermatozoa and seminal plasma in Murrah buffalo, with minimal intra-group variation and strong inter-group separation, underscoring their distinct molecular compositions.

Protein-protein interaction (PPI) analysis via the STRING database focused on spermatozoa proteins associated with reproductive function (Table 1). Zona pellucida binding protein (ZPBP) emerged as a key molecule implicated in acrosome assembly (GO:0001675) and spermatid development (GO:0007286). ZPBP was one of only three proteins associated with acrosome assembly from 20 reproductive-related proteins, with a highly significant false discovery rate (FDR) of 0.0021. Additionally, ZPBP was functionally linked to sexual reproduction and acrosomal vesicle formation, reinforcing its biological importance in fertilization. Based on STRING analysis, the protein-protein interaction network identified ZPBP as a central node with strong associations to acrosomal and sperm-binding proteins, including ACRBP, SPACA1, and SPA17, underscoring its pivotal role in sperm-oocyte recognition and fertilization (Fig 5). This finding indicates that ZPBP functions as a key regulator within the sperm proteome, potentially serving as a biomarker of fertilization competence. As shown in Fig 6, STRING interaction analysis showed that spermatozoa proteins (Fig 6A) clustered mainly around cytoskeletal components, while seminal plasma proteins (Fig 6B) were enriched in chaperones and translational machinery supporting sperm maturation. Principal component analysis (PCA) and as shown in Fig 7, multivariate analyses were performed to compare the proteomic profiles of seminal plasma and spermatozoa in Murrah buffalo. Partial least squares discriminant analysis (PLS-DA) were conducted to compare the protein profiles of seminal plasma and spermatozoa in Murrah buffalo. PCA (Fig 7A) revealed clear clustering of seminal plasma (MPL1, MPL2, MPL3; Murrah Seminal Plasma) and spermatozoa (MSP1, MSP2, MSP3; Murrah Spermatozoa) with minimal intra-group variation, while PLS-DA (Fig 7B) further emphasized the strong inter-group separation, confirming distinct proteomic compositions between the two fractions.

**Table 1:** STRING analysis of spermatozoa proteins related to reproduction.

Protein	GO-term	Protein Function	Count in network	False discovery rate	Interaction proteins
ZPBP	GO:0001675	Acrosome assembly (BP)	3 of 20	0.0021	ZPBP, ACRBP, SPACA
	GO:0007286	Spermatid development (BP)	5 of 602	0.0047	ZPBP, ACRBP, ROPN1
	GO:0019953	Sexual reproduction (BP)	4 of 151	0.0140	ZPBP, ACRBP, SPACA1, ROPN1, SPA17
	GO:0001669	Acrosomal vesicle (CC)	3 of 92	0.0223	ZPBP, ACRBP, SPACA1

**Note:** *BP: Biological Process; CC: Cellular Component.*

**Fig 8:**
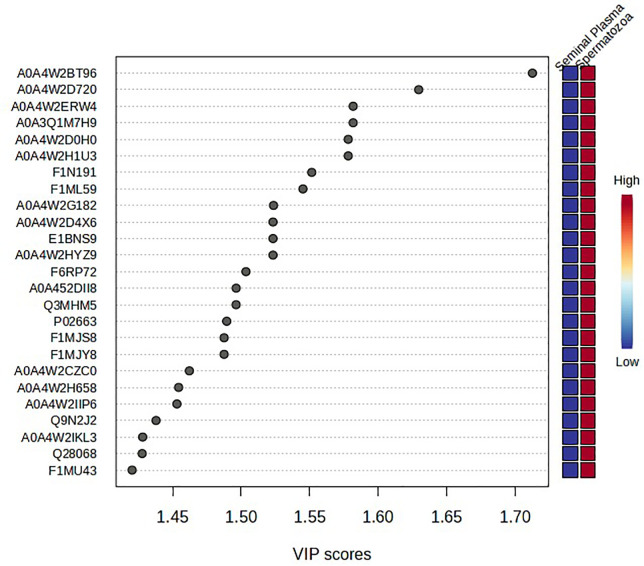
Variable importance in projection (VIP) scores for seminal plasma and spermatozoa proteins in Murrah buffalo bulls. VIP analysis identified several proteins with high discriminative power (VIP > 1.5) that contribute significantly to the proteomic divergence between seminal plasma and spermatozoa in Murrah buffalo, indicating fraction-specific molecular signatures relevant to bull reproductive function.

**Fig 9:**
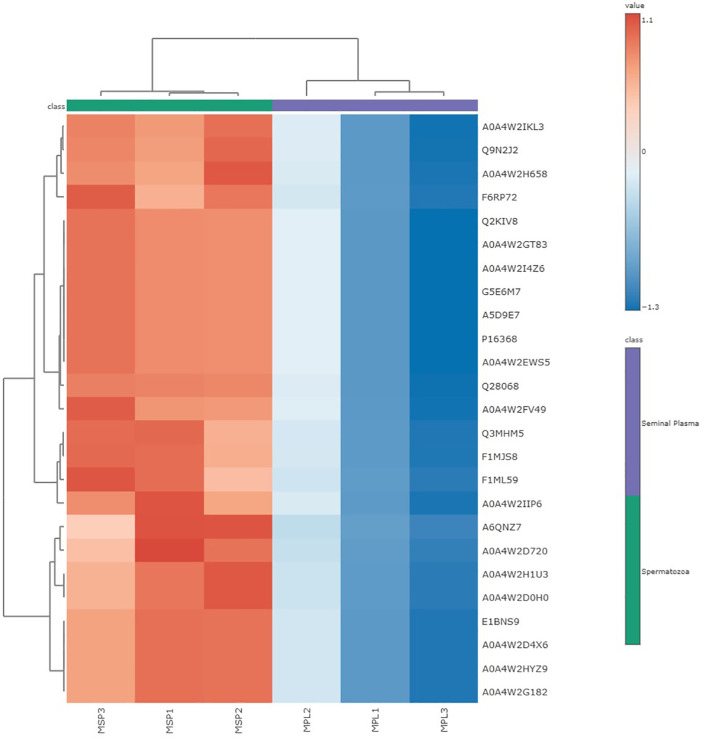
Hierarchical clustering heatmap of spermatozoa proteins in Murrah buffalo bulls across different age groups. The heatmap illustrates a pronounced segregation between spermatozoa (MSP1–MSP3) and seminal plasma (MPL1–MPL3) based on differential protein expression profiles. Distinct hierarchical clustering and expression gradients highlight fraction-specific proteomic signatures, with numerous proteins exhibiting elevated expression in spermatozoa and reduced abundance in seminal plasma, underscoring the molecular compartmentalization and functional specialization of each seminal fraction.

As presented in Fig 8, variable importance in projection (VIP) analysis identified 25 discriminative proteins between seminal plasma and spermatozoa (VIP > 1), with those exceeding a VIP score of 1.5 showing markedly higher abundance in spermatozoa. This pattern indicates spermatozoa-specific proteomic components predominantly drive the separation between the two fractions. The most significant proteins were ATP synthase subunit alpha (A0A4W2BT96), A6QNZ7, and IZUMO4 (A0A4W2D720), suggesting their central role in sample differentiation. Heatmap analysis further demonstrated distinct differences in protein expression profiles between seminal plasma and spermatozoa in Murrah buffalo (Fig 9). Proteins such as IZUMO4, A0A4W2HU3, and A0A4W2DL6 were highly abundant in seminal plasma but showed reduced expression in spermatozoa. In contrast, proteins including Q9N2W2 and A0A4W2IKL3 exhibited elevated expression in spermatozoa while being minimally expressed in seminal plasma. The hierarchical clustering clearly separated spermatozoa (MSP1, MSP2, MSP3) from seminal plasma (MPL1, MPL2, MPL3), reflecting fraction-specific grouping. The color scale represent normalized protein abundance, with red indicating higher expression and blue indicating lower expression. These findings highlight the presence of distinct proteomic signatures that underscore the molecular divergence and functional specialization between seminal plasma and spermatozoa.

## 4. Discussion

This study identified 1,893 and 1,913 proteins in the seminal plasma and spermatozoa, respectively, of Murrah buffalo bulls (Table 2), highlighting the extensive proteomic complexity of buffalo reproductive tissues. These results align with prior studies that reported a wide array of proteins—up to 2,147 in spermatozoa and 864 in seminal plasma—demonstrating the intricate molecular architecture underlying buffalo bull fertility [23]. Many identified proteins underscore the multifaceted biochemical environment necessary for supporting spermatozoa viability, motility, protection, and fertilization capacity [23,24].

**Table 2:** Identified proteins in seminal plasma and spermatozoa of Murrah buffalo bulls.

Protein	Murrah buffalo bulls (n = 8)	
	Seminal plasma	Spermatozoa
Proteome discover 2.2	450	526
Proteins selected	232	231

**Note:** *Proteome Discoverer 2.2: Proteins detected from software analysis; Selected proteins: Proteins filtered based on duplicate elimination, unique protein count (≥2), and HT score (>1).*

The functional diversity of the detected proteins suggests their involvement in essential biological processes, including energy metabolism, structural support, motility regulation, and cellular stress response—all of which are crucial for optimal spermatozoa function [18,24]. Previous studies have shown that specific proteins in the binder of sperm proteins (BSP) family correlate with semen quality traits such as motility and viability in buffalo, reinforcing the relevance of seminal plasma proteomics in fertility assessment [25]. The distinct and overlapping protein profiles between seminal plasma and spermatozoa indicate shared and specialized physiological functions. These findings provide a valuable framework for identifying candidate biomarkers and tailoring reproductive management strategies specific to Murrah buffalo bulls [24].

Seminal plasma proteins, originating primarily from the testis, epididymis, and accessory sex glands, play critical roles in modulating spermatozoa function, protecting against oxidative stress, and enhancing fertilization potential [18,26]. Conversely, sperm-specific proteins are more directly associated with structural integrity, motility, and the ability to undergo successful fertilization [27]. Most proteins identified in this study were enriched in pathways related to spermatogenesis, energy metabolism, capacitation, acrosome reaction, and sperm-oocyte interaction, supporting their roles in reproductive competence [28].

The molecular weight distribution of proteins (11.7–248.8 kDa in seminal plasma and 4.5–180.7 kDa in spermatozoa) was consistent with prior research, which identified prominent protein fractions at approximately 25.8 kDa and 58 kDa, linked to spermatozoa motility and viability in Murrah buffalo [18]. The distinct molecular weight (MW) distributions and limited overlap in identity reflect the compartment-specific roles of these proteins. Studies by Gwathmey et al. [29] and Almadaly et al. [30] reported that seminal plasma proteins in fertile bulls—particularly those of 14, 15, 26, and 30 kDa—are strongly associated with high fertility. Similarly, in indigenous Indonesian breeds, proteins ranging from 40 to 180 kDa have been correlated with improved spermatozoa quality traits [8,15,31].

The relatively low abundance of proteins with molecular weights under 10 kDa or above 200 kDa may be due to biological constraints on protein size and limitations in detection by SDS-PAGE and mass spectrometry [26,27]. Moreover, some proteins remained unclassified, likely due to the reliance on *Bos taurus* annotations for protein identification, emphasizing the urgent need for a dedicated *Bubalus bubalis* proteome database [13,32]. Functional annotation revealed a predominance of binding and catalytic activities, characteristic of proteins involved in sperm capacitation, membrane fusion, enzymatic activity, and protection against oxidative stress [33–35].

Binding-related proteins were highly prevalent in spermatozoa, implicating them in ligand interactions, structural recognition, and signal transduction, all of which are central to the acrosome reaction and zona pellucida binding during fertilization [36,37]. Among these, fertility-associated proteins such as ZPBP were particularly notable. Our findings confirmed the abundant presence of ZPBP in Murrah buffalo spermatozoa, consistent with previous studies in river buffalo, which showed that ZPBP facilitates sperm–zona pellucida binding and penetration [28].

ZPBP was also implicated in key reproductive processes, including acrosome biogenesis, spermatid development, and sperm–egg interaction. Disruption of ZPBP has been shown to impair acrosomal structure and compromise sperm–egg fusion, reducing fertilization success [38,39]. Importantly, ZPBP was functionally linked with other reproductive proteins, including ACRBP, SPACA1, ROPN1, and SPA17, forming a protein interaction network critical for acrosome reaction and sperm morphogenesis [40].

Acrosin binding protein (ACRBP) was identified as a ZPBP-interacting partner. Although its role in buffalo is less explored, studies in porcine and bovine species have demonstrated ACRBP’s involvement in capacitation, acrosome stability, and zona pellucida interaction [41,42]. Its presence in this dataset supports the hypothesis that ACRBP may also regulate acrosomal function in buffalo bulls. Sperm acrosome associated 1 (SPACA1), another key acrosomal protein, has been recognized as a molecular indicator of fertility. Low expression of SPACA1 has been linked to poor semen quality and reduced conception rates in bulls [10,43,44]. In Bali bulls (*Bos javanicus*), SPACA1 localization to the anterior acrosome suggests a role in acrosomal integrity and membrane organization during sperm maturation and fertilization.

Sperm Protein Associated with the Acrosome 17 (SPACA17) has been reported to be more abundant in sperm from high-fertility buffalo bulls [45]. Although its function is less well defined in cattle, SPA17 is associated with oxidative phosphorylation and motility in bulls and is thought to mediate zona pellucida binding during fertilization [46]. Rhophilin-1 (ROPN1) was also detected in our analysis, a protein that maintains the fibrous sheath’s structural integrity and regulates sperm motility [47]. Its presence in this network further supports its physiological relevance to buffalo fertility. The detection of metabolic enzymes such as lactate dehydrogenase C (LDHC) reaffirms the importance of energy metabolism in supporting sperm motility and fertilization potential [48]. However, our study was limited by the absence of fertility-stratified groups, which restricts the direct correlation between protein expression and fertility outcomes. Future studies should incorporate high- and low-fertility cohorts to validate differentially expressed proteins using methods such as 2D-SDS-PAGE, immunoblotting, or targeted proteomics.

## 5. Conclusion

This study provides a comprehensive proteomic characterization of seminal plasma and spermatozoa in Murrah buffalo bulls, revealing a diverse array of proteins involved in critical reproductive processes. Notably, proteins such as ZPBP, ACRBP, SPACA1, SPA17, and ROPN1 emerged as promising fertility-associated biomarkers, implicated in acrosome formation, sperm motility, zona pellucida binding, and overall fertilization competence. These findings enhance the molecular understanding of buffalo bull fertility and establish a solid foundation for the development of diagnostic tools to support more accurate bull selection and fertility assessment in reproductive management programs.

However, this study has certain limitations. The sample size was relatively limited and focused exclusively on a single breed, which may not fully capture the proteomic diversity present across different buffalo populations. Expanding the sample cohort and including other buffalo breeds would enrich the dataset and improve the generalizability of the findings. Furthermore, in-depth investigation of the dominant proteins identified—particularly those with strong associations to reproductive function—will be critical for validating their utility as reliable, species-specific biomarkers. Future research focusing on these key proteins using targeted proteomic and functional validation approaches will significantly advance the discovery of protein-based biomarkers for buffalo fertility, ultimately contributing to the improvement of artificial insemination outcomes and the genetic progress of buffalo herds worldwide.
